# The Physical Properties and Crystallization Kinetics of Biocomposite Films Based on PLLA and Spent Coffee Grounds

**DOI:** 10.3390/ma15248912

**Published:** 2022-12-13

**Authors:** Jan Novák, Luboš Běhálek, Martin Borůvka, Petr Lenfeld

**Affiliations:** Faculty of Mechanical Engineering, Technical University of Liberec, Studentská 1402/2, 46117 Liberec, Czech Republic

**Keywords:** biocomposites, poly(L-lactic acid), spent coffee grounds, itaconic anhydride, plasticizer

## Abstract

In the context of today’s needs for environmental sustainability, it is important to develop new materials that are based on renewable resources and biodegrade at the end of their life. Bioplastics reinforced by agricultural waste have the potential to cause a revolution in many industrial applications. This paper reports the physical properties and crystallization kinetics of biocomposite films based on poly(L-lactic acid) (PLLA) and 10 wt.% of spent coffee grounds (SCG). To enhance adhesion between the PLLA matrix and SCG particles, a compatibilizing agent based on itaconic anhydride (IA)-grafted PLLA (PLLA-g-IA) was prepared by reactive extrusion using dicumyl peroxide (DCP). Furthermore, due to the intended application of the film in the packaging industry, the organic plasticizer acetyl tributyl citrate (ATBC) is used to improve processing and increase ductility. The crystallization behavior and thermal properties were observed by differential scanning calorimetry (DSC) and thermogravimetric analysis (TGA). Crystallinity degree increased from 3,5 (neat PLLA) up to 48% (PLLA/PLLA-g-IA/ATBC/SCG) at the highest cooling rate. The physical properties were evaluated by tensile testing and dynamic mechanical analysis (DMA). The combination of the compatibilizer, SCG, and ATBC led to a synergistic effect that positively influenced the supramolecular structure, internal damping, and overall ductility of the composite films.

## 1. Introduction

The invention of catalytic polymerization caused massive petroleum-based plastics production that presents a significant challenge for the environmental sustainability [1,2,3]. The bio-based materials made from renewable resources have the potential to be commercially and environmentally acceptable. Especially in Europe, attention to the production of greenhouse gas emission, renewability, and toxicity is given, and the market with these environmentally friendly materials is growing fast [4,5]. Currently, one of the most commonly used biopolymers is poly(lactic acid) (PLA) [6]. Due to its biocompatibility and bioresorbability, PLA was initially used for medical purposes, e.g., bone fixation [7]. More affordable production and the possibility of biodegradation through the industrial composting process increased PLA application potential also to packaging [8], textiles [9], molding [10], and additive technologies [11]. PLA is a linear aliphatic thermoplastic polyester with high modulus and tensile strength and excellent barrier properties [12]. By contrast, its disadvantages include brittleness [13], a low heat deflection temperature [14], crystallization [15], and a biodegradation rate [16]. Many material modifications could be made to enhance these properties. Blending with other polymers, copolymerization, and the addition of particulate fillers and fibers or plasticizers are commonly used methods [17,18,19,20,21]. For example, adding the organic plasticizers, triethyl citrate (TC) and acetyl-tributyl citrate (ATBC) are used to enhance ductility due to the increase in chain mobility while maintaining biodegradability [22]. Other PLA usage limits, such as low heat deflection temperature and mechanical properties, can be improved through the enhancement of crystallization kinetics [23,24]. One option is to increase the nucleation density by adding heterogeneous nucleation agents [25,26]. The simultaneous addition of the nucleation agents and plasticizers may results in a synergistic effect, which can positively affect the crystallinity degree of PLA [27]. In last decades, many studies have investigated the use of fiber [28] and micro or nano particulate fillers [29,30] and their potential use as nucleating agents. Currently, the natural bio-based fillers, such as hydroxyapatite [31], cellulose nanocrystals [32], biochar [33], rice husks [34], or spent coffee grounds [35], are used. Spent coffee grounds containing cellulose, hemicellulose, lignin, and coffee oil, due to its composition, preferably act as a plasticizer and filler to improve the mixing process [36,37,38]. For example, Suaduang et al. [38] found a 33% increase in MFR for PLLA containing 10% of spent coffee grounds. Another promising usage of agricultural waste is to obtain new environmental-friendly polymeric material by liquification [39]. Furthermore, due to the lack of PLA-reactive side chain groups, surface and bulk modification is challenging, which can cause poor adhesion in composite structures [40,41]. Among chemical modification possibilities, free radical grafting is arguably one of the most successful and cost-effective treatments for preparing compatibilizers and improving interface adhesion. A number of studies was published for effect assessment of silanes, styrenes, or maleic anhydride (MAH) as compatibilizing agents [41,42,43]. Using MAH in PLA composites with low filler concentrations shows also significant impact on the degree of crystallinity, especially at slow cooling rates. Běhálek et al. [44] observed an increase in the absolute degree of crystallinity (X_c_) from 54.4 to 60.1% for PLA with 10 wt.% of cellulose fibers by the grafting MAH onto a PLA backbone. However, due to the poor activity of MAH toward macro-radicals, the grafting efficiency is low compared to other polyesters [45]. Additionally, the reaction of MAH-grafted polymers with proteins may result in unstable amid bonds that can easily be hydrolyzed [40]. Itaconic anhydride (IA), with its chemical similarity, represents an alternative to MAH, and moreover, it is derived from renewable resources [46]. The high degree of grafting was reached by Verbeek et al. [47] for polyethylene. Crosslinking during the grafting reactions reduced elongation at break while improving the impact strength, creep resistance and not affecting the tensile strength [48]. Ku Marsilla et al. [40] found an increase in elongation at break, tensile strength, and energy to break for PLA grafted by 5 wt.% IA. The main aim of this study was to evaluate the thermal, mechanical, and structural properties of prepared bio-composite structures, where the reactive extrusion of itaconic anhydride with PLA was applied to reduce the interfacial tension between the matrix and spent coffee grounds (SCG) filler. Due to the brittleness of the PLA and in the view of the intended application, an organic plasticizer, ATBC, was incorporated into a bio-composite. A variety of characterization methods were used, such as Fourier transform infrared spectroscopy (FT-IR), scanning electron microscopy (SEM), differential scanning calorimetry (DSC), thermogravimetric analysis (TGA), tensile test, and dynamic mechanical analysis (DMA). Furthermore, the effect of SCG, ATBC, IA grafting onto PLA chain, and cooling rate of the non-isothermal crystallization process on the crystallization kinetics was investigated in detail using a modified Avrami equation.

## 2. Materials and Methods

Poly(L-lactic acid) (PLLA) matrix under the trade name of Luminy L130 was supplied by Total Corbion PLA (Gorinchem, The Netherlands). It has an approximate molar mass of 170,000 g/mol, a glass transition temperature (T_g_) of 60 °C, a melting point of 175 °C, and contains more than 99% of L-lactide. Particulate spent coffee ground filler (SCG) Kimbo 100% Arabica (De’Longhi S.p.A., Treviso, Italy) was ground to an average particle size lower than 20 µm. In addition to that, 95% itaconic anhydride (IA) (Sigma-Aldrich, Darmstadt, Germany) with an approximate molar mass of 112 g/mol in the form of a powder as a compatibilizer agent and 98% dicumyl peroxide (DCP) (Sigma-Aldrich, Darmstadt, Germany) with an approximate molar mass of 270 g/mol as an initiator was used. Dehydrated acetone (ACROS Organics, Geel, Belgium) was applied to dissolve DCP and IA. A liquid form of ATBC, Citroflex A-4 delivered by Vertellus Holding LLC (Indianapolis, IN, USA), was used as a plasticizer.

### 2.1. Masterbatch Preparation and Film Processing

For the bio-composite film extrusion, at first, it was necessary to prepare masterbatches (MB) (Table 1), which were combined with PLLA pellets to reach the targeted compositions (Table 2). All additives, as well as the PLLA, were dried in vacuum oven VD53 (Binder GmbH, Tuttlingen, Germany) to remove moisture for 24 h at 60 °C. For the MB1 preparation, IA and DCP were dissolved in 50 mL of dehydrated acetone and added to PLLA. Further, the material was dried again and reactive extrusion with a subsequent granulation process was carried out. As a processing device, the laboratory micro-compounder MC 15 HT (Xplore, Sittard, The Netherlands) with conical screws, a constant temperature profile of 180 °C and a screw speed of 60 rpm was used. In this study, the IA and DCP concentration was fixed to 6 and 0.5% according to conclusions of Ku Marsilla et al. [40]. The dispersion and distribution were controlled by the level of torque. Consequently, the dried bio-composite pellets were processed on the twin-screw extruder MC 15 HT with a flat film die (210 °C, 0.2 mm gap size) at a melt temperature of 200 °C and 60 rpm screw speed. The extruded films were drawn by air-cooled rolls.

### 2.2. Spectral Analysis

To study the grafting of IA onto the PLLA, the Fourier transform infrared spectroscopy (FT-IR) using a Nicolet iS10 (Thermo Fisher Scientific, Waltham, MA, USA) spectrometer was performed. Measurements were taken with single-reflection attenuated total reflectance (ATR) system equipped with a diamond crystal. The specimens were dried in a vacuum oven VD53 at 60 °C for 24 h before the characterization. Each measurement consisted of 32 scans with 4 cm^−1^ resolution in the 400 to 4000 cm^−1^ range.

### 2.3. Scanning Electron Microscopy (SEM)

The morphology of fractured surfaces was examined by field emission scanning electron microscopy (FE-SEM) using the TESCAN MIRA 3 (Tescan, Brno, Czech Republic) instrument with an accelerated voltage of 5 kV. The film specimens were frozen in liquid nitrogen (−196 °C) and crushed. The fractured surfaces were coated with 5 nm of gold using a sputter coater LEICA EM ACE 200 (Leica Microsystems, Wetzlar, Germany).

### 2.4. Crystallization Kinetics

The non-isothermal study of crystallization kinetics was conducted by differential scanning calorimetry (DSC) analysis using a DSC 1/700 (Mettler Toledo, Greifensee, Switzerland) at different cooling rates at 5 to 40 °C min^−1^. The facility was calibrated via zinc and indium standards. For each measurement, it was prepared about 4–8 mg of the film sample, and an empty pan was used as a reference. The samples were heated from 0 to 200 °C with a heating rate of 10 °C min^−1^ and performed under a constant nitrogen flow of 50 mL min^−1^. The following thermal properties were observed for each composite film: the enthalpy of melting (ΔH_m_), the enthalpy of cold crystallization (ΔH_cc_), and the enthalpy of pre-melt crystallization (ΔH_pc_). In addition to that, the degree of crystallinity (X_c_) was determined according to the following equation:

(1) $$X_{c\;}=\frac{∆H_{m}-∆H_{\mathrm{cc}}-∆H_{\mathrm{pc}}}{∆H_{m}^{0}\cdot w_{m}}$$

where (ΔH_m_^0^) is the melting enthalpy of 100% crystalline PLLA (106 J g^−1^) [49] and w_m_ is the mass content of PLLA in the composites.

Further, the melting temperature (T_m_), cold crystallization temperature (T_cc_), pre-melt crystallization temperature (T_pc_), melt crystallization temperature (T_c_), and enthalpy of melt crystallization (ΔH_c_) were tracked. Relative crystallinity (X_T_) was calculated according to the following equation:

(2) $$X_{T\;}=\frac{∆H_{T}}{∆H_{C}}=\frac{\int_{T_{0}}^{T}\left(\frac{{\mathrm{dH}}_{c}}{\mathrm{dT}}\right)\mathrm{dT}}{\int_{T_{0}}^{T^{\infty }}\left(\frac{{\mathrm{dH}}_{c}}{\mathrm{dT}}\right)\mathrm{dT}}$$

where (ΔH_T_) is the heat of melt crystallization, (T_1_) initial temperature, (T) temperature at a given moment, and (T^∞^) final temperature. The melt crystallization time (t_c_) was determined by the following equation:

(3) $$t_{c\;}=\frac{T_{0}-T}{\mathrm{CR}}$$

where (CR) is the cooling rate.

Jeziorny-modified model of the logarithmic transformation of Avrami Equation (4) was used to consider the temperature changes during the crystallization process [50,51]. The growth rate parameter (Z_t_) is corrected for CR, giving a modified growth rate constant (Z_c_), as follows:

(4) $$\log\left(-\ln\left(1-X_{T}\right)\right)=\log Z_{t}-n\log t$$

(5) $$\log Z_{c}=\frac{\log Z_{t}}{\mathrm{CR}}$$

### 2.5. Thermal Degradation

The temperature of thermal decomposition of each additive and matrix was set by thermogravimetric analysis (TGA) using TGA2 instrument (Mettler Toledo, Greifensee, Switzerland). The samples were heated from 50 to 600 °C under N_2_ atmosphere at the heating ramp of 10 °C min^−1^. The initial decomposition temperature was determined at 5% mass loss. Prior to the measurements, the temperature sensor was calibrated using the Curie temperature of ferromagnetic materials based on isotherm, trafoperm, and nickel alloy.

### 2.6. Mechanical Measurement

Measurement of mechanical properties was performed by using a TIRA test 2300 (Tira, Schalke, Germany) testing machine equipped with a load cell of 1 kN and extensometer MFX 500-B (Mess & Feinwerktechnik GmbH, Velbert, Germany). Measurements have been performed according to the ISO 527 standard. Young’s modulus measurements were performed at a crosshead speed of 1 mm min^−1^ and tensile strength with elongation at break at a crosshead speed of 5 mm min^−1^. According to ISO 291 standard, all the specimens were conditioned in a KSP climatic chamber (Teseco, Kostelec nad Orlici, Czech Republic) at 23 °C and 50% relative humidity for one week.

### 2.7. Dynamic Mechanical Analysis (DMA)

The measurement of dynamic mechanical properties was carried out on the DMA DX04T (RMI s.r.o., Lazne Bohdanec, Czech Republic) equipment with the constant sinusoidal strain under the temperature of 0 to 80 °C, frequency of 1 Hz, and deformation amplitude of 0.2 mm. The square specimens (10 × 10 mm) were obtained from the films and tensile tested. The heating rate was 2 °C min^−1^, and the storage modulus (E′), loss modulus (E″), complex modulus (E*), and loss factor (tan δ) were evaluated according to Equations (6)–(9). The values of these parameters were determined at temperatures of 10, 23, 40, and 50 °C.

(6) $$E^{\prime }=E^{*}\;.\;\cos\delta$$

(7) $$E^{\prime \prime }=E^{*}\;.\;\sin\delta$$

(8) $$\tan\delta =\frac{E^{\prime \prime }}{E^{\prime }}$$

(9) $$E^{*}=E^{\prime }+\;i\;.E^{\prime \prime }$$

## 3. Results

### 3.1. Spectral Analysis

**Figure 1:**
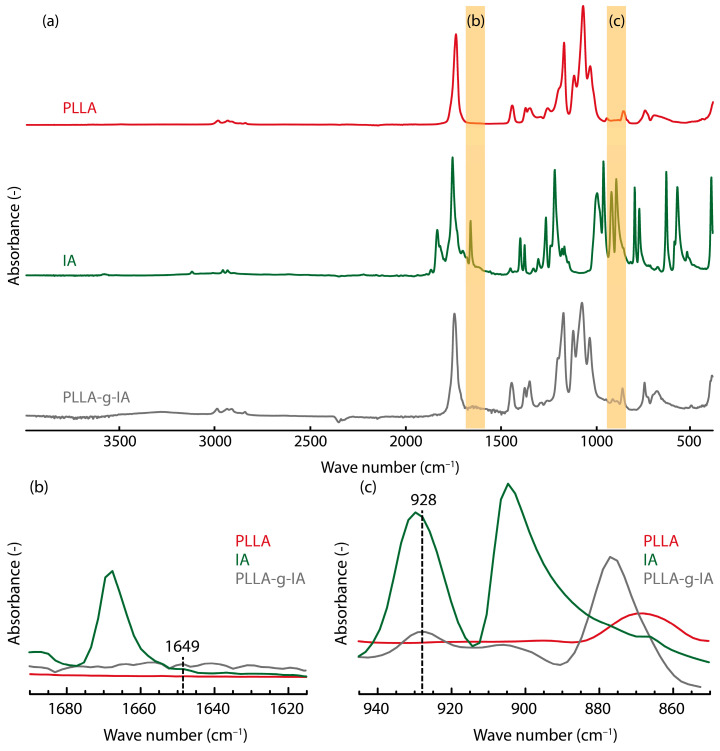
FT-IR spectra of PLLA Luminy L130, IA, and PLLA-g-IA: (**a**) full FT-IR spectrum from 4000 cm^−1^ to 400 cm^−1^, (**b**) absorbance of the C=C stretching at 1649 cm^−1^, and (**c**) absorbance of the C-O-C stretching at 928 cm^−1^.

The presence of IA on the PLLA chain was identified through FT-IR measurements, which were compared to neat PLLA and IA (Figure 1). Full spectra of each component are shown in Figure 1a. As shown in Figure 1b,c, new absorption bands appear at 1649 cm^−1^ and 928 cm^−1^ in the spectra of PLLA-g-IA. A new peak can be observed at 1649 cm^−1^ for PLLA-g-IA, which is not noticeable for a neat PLLA sample (Figure 1b). This absorption was ascribed to the stretching vibrations of the cyclic anhydride functional group (C=C), which prove the mechanism of IA grafting onto PLA by Ku Marsilla [40]. Absorption band at around 2920 cm^−1^ can be assigned to CH_2_ functional groups, which could be considered as proof of grafted IA since CH_2_ groups are included in its molecule [52]. A peak reflecting C=O stretching vibration of an anhydride ring was probably not observed due to the low concentration of grafted IA [46]. Absorption band at 928 cm^−1^ can be ascribed to the symmetric stretching vibration of the C-O-C functional group (Figure 1c). This conclusion confirms successful grafting of IA monomers onto the PLLA backbone.

### 3.2. Scanning Electron Microscopy (SEM)

**Figure 2:**
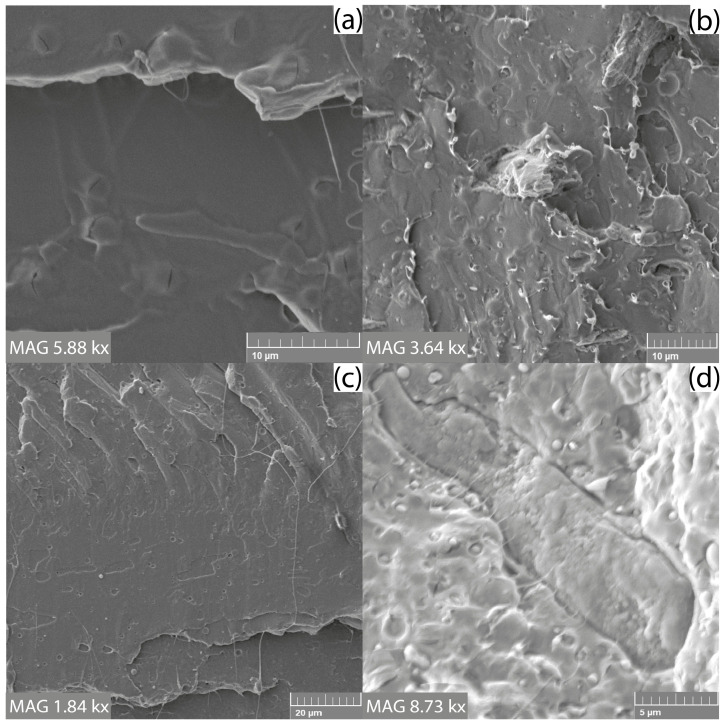
SEM analysis of fractured surfaces: (**a**) PLLA, (**b**) PLLA/SCG, (**c**) PLLA/PLLA-g-IA, and (**d**) PLLA/PLLA-g-IA/SCG.

**Figure 3:**
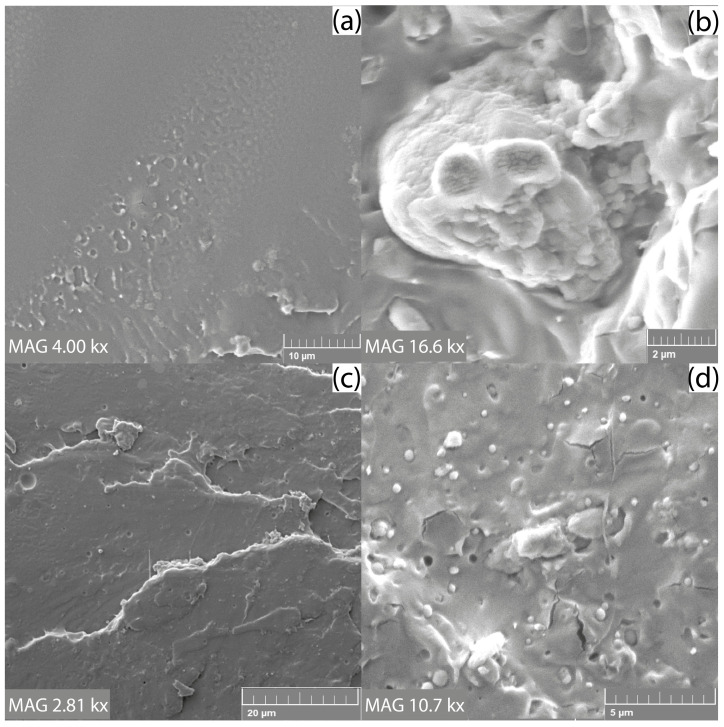
SEM analysis of fractured surfaces: (**a**) PLLA/ATBC, (**b**) PLLA/ATBC/SCG, (**c**) PLLA/PLLA-g-IA/ATBC, and (**d**) PLLA/PLLA-g-IA/ATBC/SCG.

Structural analysis of the fractured surfaces of the composite films using electron microscopy was performed to assess the effect of the compatibilizing agent (IA) and plasticizer (ATBC) on the interfacial adhesion between the SCG and the PLLA matrix. The brittle fracture of all studied films could be observed in the microscopic images. In this case, Figure 2b shows that the adhesion between the hydrophobic matrix and the hydrophilic natural filler is sufficient even without the use of a compatibilizing agent. However, with the addition of PLLA-g-IA, SCG particles were coated and bonded to the matrix better than in the PLLA/SCG composite (Figure 2d). The reduction in the interfacial tension between the filler and matrix should be mainly reflected in the mechanical properties of the films. For the sample PLLA/ATBC/SCG, the interfacial adhesion is also at a very good level, which may be caused by higher chain mobility due to the addition of a plasticizer that contributed to better envelopment of the SCG filler (Figure 3b). The same results were observed for PLLA/PLLA-g-IA/ATBC/SCG composite films (Figure 3d).

### 3.3. Crystallization Kinetics

**Figure 4:**
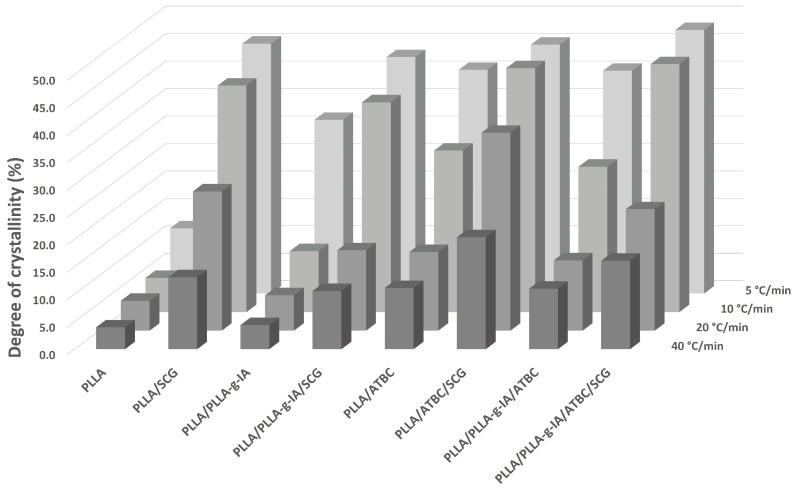
Crystallinity degree of biocomposite structures at different CRs.

The extruded film samples were studied using DSC analysis according to the ISO 11,357 standard to specify morphology and thermal properties. In this section, the results obtained from the melt cooling run and the second heating run, which determine the crystallization capability of the materials, are discussed. In terms of the transition temperatures, the results of the DSC analysis show that the melting temperature of the composite structures is mainly determined by the polymer matrix in range of 172 ± 2 °C, see Table 3. In the presence of ATBC, the melting temperature slightly decreases to values of 168 ± 2 °C. As expected, the effect of ATBC is particularly pronounced in changing the T_g_, which is in range of 45 ± 5 while for PLLA material structures without ATBC in range of 59 ± 2 °C. However, the determination of the T_g_ by DSC is affected by a methodological error, which is particularly visible at lower cooling rates, when the primary crystallization of the polymer system is greatest, increasing its degree of crystallinity and resulting in difficulty in determining the glass transition region. The study of crystallization kinetics has shown, in addition to well-known facts, such as the effect of CR on the X_c_ and a positive effect of SCG on the formation of the PLLA crystalline structure, see Figure 4. In contrast, no positive effect of IA on the X_c_ of the PLLA/SCG composite structure was demonstrated, although grafting IA onto PLLA chain alone resulted in an increase in X_c_. Comparing the PLLA and PLLA/PLLA-g-IA matrices, an increase in crystallinity of 2.6 times can be found at CR of 5 °C/min. In contrast, the comparison of the PLLA/SCG and PLLA/PLLA-g-IA/SCG composites shows a 5% decrease in crystallinity, which could be observed at all investigated CRs. Thus, it can be concluded that the grafting of IA onto the PLLA chain does not achieve a synergistic effect in the crystallization of SCG composite structures, whereas in the case of the PLLA/ATBC/SCG, it is noticeable, and an increase in the X_c_ was observed at all CRs.

A positive finding is that the increase in X_c_ is particularly evident even at high CRs, which otherwise generally hinder polymer crystallization. At slow melt CR (5 °C/min), the PLLA/PLLA-g-IA/ATBC/SCG achieved the highest value of X_c_ = 48%, showing an increase of 18% compared to PLLA/PLLA-g-IA/ATBC and a fourfold increase compared to neat PLLA matrix. A similar result can be observed for the PLLA/SCG structure. At higher CRs (40 °C/min), the crystallization ability of PLLA is limited. The values of X_c_ show that a higher change in the macromolecule arrangement can be achieved at higher CRs due to the SCG addition; for example, a more than threefold X_c_ increase was observed for the PLLA/SCG structure, while PLLA/PLLA-g-IA/SCG increased 2.4 times (only 1.4-fold at 5 °C/min). The highest values of X_c_ at CR = 40 °C/min were achieved by the PLLA/ATBC/SCG (X_c_ = 20%), where an increase of about 2 times was observed compared to the PLLA/ATBC matrix, while at CR = 5 °C/min this increase was only 10%. Overall, the organic plasticizer based on ATBC can be attributed to an increase in the conformational processes of macromolecules promoting the crystallization behavior of the polymer. Its addition to PLLA results in an increase in X_c_ at all CRs. The positive effect of the SCG can be clearly seen in the increase in enthalpy changes in the primary crystallization and the suppression of the secondary crystallization. The temperature of secondary crystallization (T_sc_) shifts to significantly lower values (Table 3), indicating that SCG composites require lower kinetic energy for the internal rotations of macromolecules, similar to the PLLA/ATBC structures.

**Figure 5:**
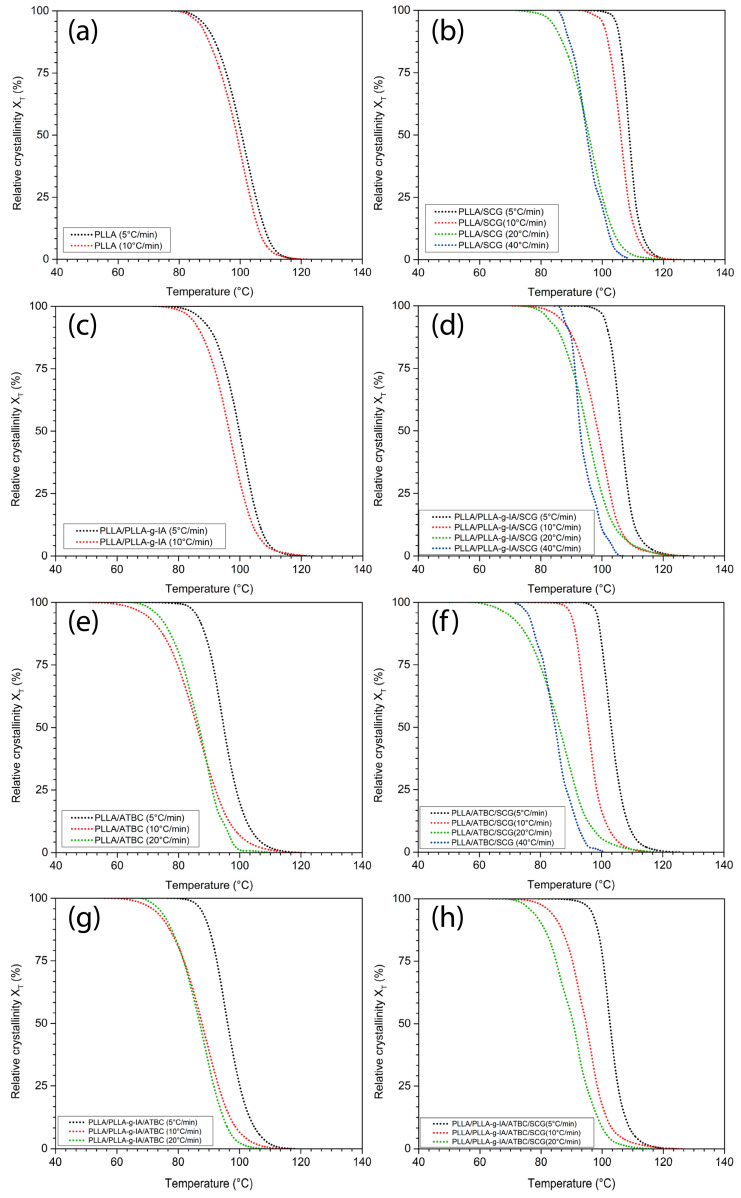
Variation in X_T_ during the cooling rates 5, 10, 20, and 40 °C/min for (**a**) neat PLLA, (**b**) PLLA/SCG, (**c**) PLLA/PLLA-g-IA, (**d**) PLLA/PLLA-g-IA/SCG, (**e**) PLLA/ATBC, (**f**) PLLA/ATBC/SCG, (**g**) PLLA/PLLA-g-IA/ATBC, and (**h**) PLLA/PLLA-g-IA/ATBC/SCG.

**Figure 6:**
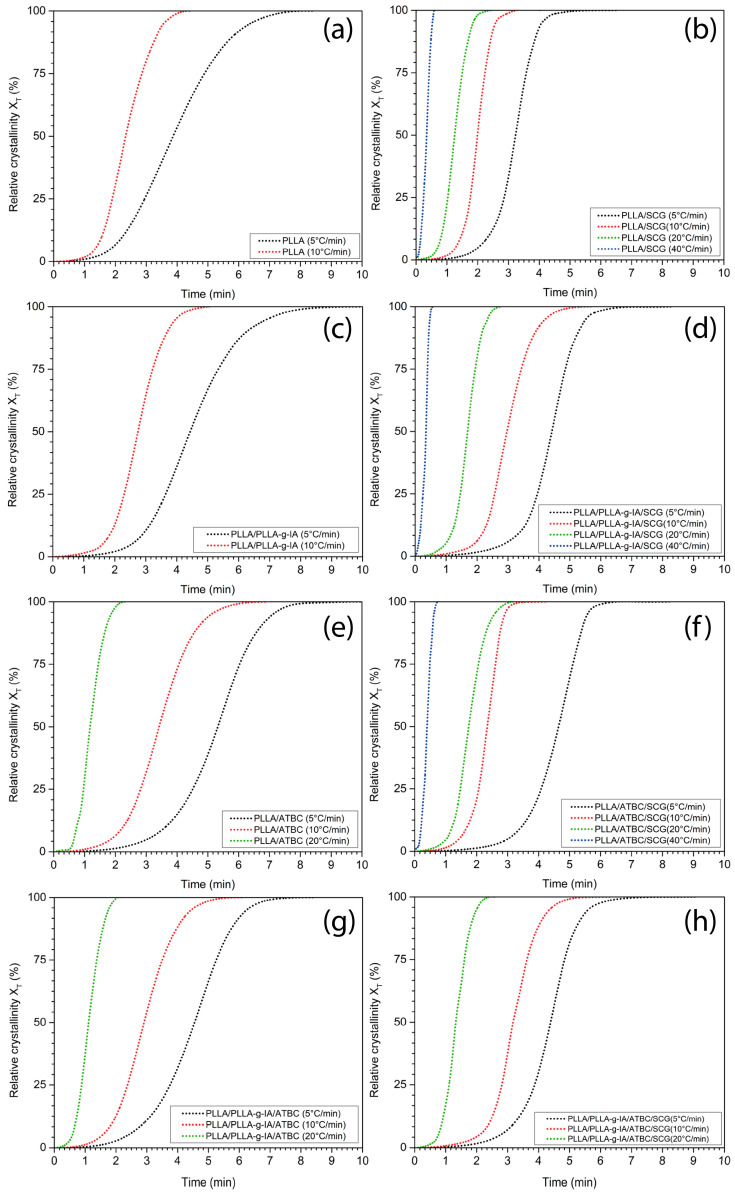
Plots of X_T_ as a function of time during the cooling rates 5, 10, 20, and 40 °C/min for (**a**) neat PLLA, (**b**) PLLA/SCG, (**c**) PLLA/PLLA-g-IA, (**d**) PLLA/PLLA-g-IA/SCG, (**e**) PLLA/ATBC, (**f**) PLLA/ATBC/SCG, (**g**) PLLA/PLLA-g-IA/ATBC, and (**h**) PLLA/PLLA-g-IA/ATBC/SCG.

Such as cellulose nanocrystals [26], lignin nanoparticles [53] or cellulose fibers, and rice husks [44], SCG act as a nucleating agent to promote overall crystallization of PLLA. This is due to the higher onset of the crystallization temperature and the peak of the primary crystallization temperature (T_c_), respectively, see Table 4. For example, at CR of 5 °C/min, T_c_ = 101.7 °C for PLLA and 108.2 °C for PLLA/SCG. Similarly, this phenomenon can be observed for the PLLA/ATBC/SCG structure, where at the same CR, the T_c_ increased from 93.8 to 101.6 °C, see Table 4. The relative crystallization (X_T_) determined at T_c_ and halftime of the crystallization process (t_1/2_) corresponding to X_T_ = 50% were also monitored to assess the crystallization kinetics. As was mentioned above, the presence of SCG increases the primary crystallization temperature, whereas ATBC decreases it. Thus, the PLLA/SCG crystallinity half-time is smaller than PLLA/ATBC/SCG at all studied CRs. The effect of SCG presence in the PLLA, PLLA/PLLA-g-IA, PLLA/ATBC, and PLLA/PLLA-g-IA/ATBC is shown in Figure 5 and Figure 6.

An increase in the crystallization halftime also occurs when IA is grafted onto the PLLA chain. At a CR = 5 °C/min, in all composite structures, the t_1/2_ decreased due to the presence of SCG. At higher melt CRs, this trend persisted especially for the PLLA/SCG and PLLA/ATBC/SCG. For the PLLA/PLLA-g-IA/SCG and PLLA/PLLA-g-IA/ATBC/SCG, there is a slight increase in the t_1/2_. Thus, it can be assumed that grafting of IA onto the PLLA backbone suppresses the rate of crystallization. The results of the change in X_T_ clearly indicate a higher rate of the PLLA crystallization process in the presence of SCG and ATBC. Especially at lower CRs, the synergistic effect of SCG with ATBC in the PLLA matrix was confirmed (X_T_ has grown up to 52% compared to neat PLLA). These conclusions are further supported by the Jeziorny modification of Avrami crystallization kinetics model. The statistical parameters obtained by the linear fitting of the dependence of log[-ln(1-X_T_)] and log t are listed in Table 5.

The Avrami exponent n as a measure of uniform crystal growth increases by the addition of SCG from 3.25 to 4.58 at CR = 5 °C/min. The values of n could be concluded to be a mixed-type growth with variable nucleation. With the increase in the CR, the values of Z_c_ increase and t_1/2_ decreases, which indicates the higher crystallization rate with the increase in CR. Additionally, in the presence of SCG, the values of Z_c_ and t_1/2_ changed from 0.00069 and 3.23 min at CR = 5 °C/min to 0.037839 and 0.34 min at CR = 40 °C/min. Therefore, based on the results of t_1/2_ and Z_c_, we could draw the conclusion that the presence of SCG has a positive effect on PLLA crystallization kinetics, especially at higher CRs. The same conclusion was reached for PLLA/PLLA-g-IA and PLLA/ATBC matrix. The value of n is depending on the nature of the nucleation and growth geometry of crystals [54]. This indicates that the presence of SCG has a great influence on the nucleation and growth geometry of PLLA. The highest effect could be observed for PLLA/PLLA-g-IA/SCG and PLLA/ATBC/SCG, where the value of n was 2.63 and 2.96 at CR = 40 °C/min, while for the other composite structures, there was not a noticeable area of primary crystallization.

### 3.4. Thermal Degradation

The thermodegradation temperatures are summarized in Table 6. Thermal stability of neat PLLA, SCG, and ATBC reaches its limit at 328, 253, and 222 °C, respectively. Thus, the addition of ATBC into PLLA films caused a significant decrease in the initial decomposition temperature. Similar results were observed by Maiza et al. [22] and others [55,56]. Grafting IA onto the PLLA backbone slightly increased thermal stability, which is ascribed to the new C=C bond. The addition of SCG reduced the decomposition temperature to approximately 310 °C, which is related to the degradation of low molecular weight components, such as hemicellulose, that is present in the lignocellulosic residue [57]. A similar reduction in thermal stability was observed by Terroba-Delicado et al. [25] or Mendes et al. [58] when studying PE/SCG thermal properties. Nekhamanurak et al. [59] observed PLLA/CaCO_3_ fatty acid-treated composites and concluded the reaction of the fatty acids and oils with ester linkage of PLLA decreases its thermal stability. The incorporation of SCG into PLLA/PLLA-g-IA/ATBC matrix points to the synergistic effect between SCG and ATBC in the presence of PLLA-g-IA.

### 3.5. Mechanical Measurements

The results from tensile tests of neat PLLA, modified PLLA, and SCG-based composites prepared by extrusion are shown in Table 7. The measured values show that the Young’s modulus depending on the presence of SCG does not change in practice, which is positive since the moisture content of the SCG composite film 0.7–0.9% is higher than that of the neat PLLA 0.3–0.4%. The same conclusion was reached for the composite with grafted itaconic anhydride onto the PLLA chain. Significant changes are observed for the PLLA/ATBC/SCG film, where the presence of coffee grounds in the PLLA/ATBC matrix causes a decrease in tensile modulus of about 28% (from 2834 MPa to 2049 MPa) and an increase in the elongation more than five times (from 7 to 36%), although the degree of crystallinity increased by 63%. These results indicate the synergistic effect of ATBC and SCG containing fatty acids and other components reducing the stiffness and increasing the ductility of the material, which was not achieved by PLLA containing SCG and ATBC separately. This synergistic effect was also observed for the composite films PLLA/PLLA-g-IA/ATBC and PLLA/PLLA-g-IA/ATBC/SCG, respectively. The presence of SCG led to a 12% decrease in the average modulus and in an increase in ductility by approximately 90%. A comparison of the tensile test results shows that the addition of SCG to PLLA, PLLA/PLLA-g-IA, and PLLA/ATBC matrix reduces the tensile strength as expected. This effect is also supported by the increase in the elongation at break, which confirms the SCG plasticizing ability. Similar results were observed by Suaduang et al. [35] on the bio-composite PLA/SCG blown films. It was found that a greater content of SCG significantly decreases the tensile strength, and the results of elongation at break showed the reduction to brittleness compared to neat PLA. This brittleness reduction is supported by ATBC content. The ATBC with abundant polar groups easily enters the gap between PLA chains, which weakens the binding and entanglement force between PLA chains and increases their mobility [60]. Tian et al. observed that 20 wt.% of ATBC resulted in the great increase in the elongation at break while the tensile strength and tensile modulus reduce observably when compared with the 15 wt.% ATBC modified PLA, which is attributed to the reduction in intermolecular forces [60,61].

### 3.6. Dynamic Mechanical Analysis (DMA)

The experimental research has investigated the temperature dependence of the dynamic modulus of elasticity (E*), including its real (E′) and imaginary (E″) components, and the loss factor (tan δ) characterizing the internal damping of the material. The parameters determined by DMA at temperatures of 10, 23, 40, and 50 °C, respectively, are shown in Table 8. The temperature of 10 °C represents cooler conditions, the temperature of 23 °C corresponds to standard environmental conditions, the temperature of 40 °C corresponds to the mean glass transition temperature of PLLA/ATBC-based films, and the temperature of 50 °C corresponds to the glass transition of other studied composite films. With the addition of SCG, the results of the DMA show an increase in the imaginary component of the dynamic modulus, which is characterizing the damping properties of the material. The change in tan δ is insignificant as the imaginary component of the dynamic modulus increases along with its real component. This is not the case for the PLLA/ATBC/SCG film due to the content of a plasticizer, which reduces the elastic modulus. In contrast to the static tensile test, a positive effect of SCG on the elastic modulus and damping properties was observed. An increase in these properties may be promoted by the presence of fatty acids in SCG, which is in line with the conclusions of Coelho et al. [62]. The differences in the values of the elastic modulus found in static and dynamic loading are caused by the nature of the test and the range of deformation regions. The greatest effect of SCG onto damping properties was clearly observed for the PLLA/ATBC/SCG film, which corresponds to the change in the nominal relative elongation at break, see Table 7. The SCG in the given composite system increased the imaginary elastic modulus and the loss factor up to 2.9 and 3.8 times, respectively. The deviations observed for the PLLA/PLLA-g-IA/ATBC/SCG composite structure may be due not only to the frequency of the measurements but also to the frequency of the recorded data and their approximation.

## 4. Conclusions

In this paper, the itaconic anhydride (IA) was successfully grafted onto PLLA backbone by a reactive extrusion process, and consequently, acetyl tributyl citrate (ATBC) and spent coffee grounds (SCG) were incorporated. To increase the application potential, the effect of the organic plasticizer on the supramolecular structure, thermal, mechanical, and dynamic-mechanical properties of the PLLA/SCG bio-composite were investigated. The presence of SCG and ATBC caused not only an increase in elongation at break of PLLA composites but also enhanced crystallization kinetics. The positive effect of SCG was observed as the nucleation barrier in the primary crystallization region decreased. A synergistic effect occurring especially at higher cooling rates (CR) was achieved for the PLLA/ATBC/SCG composite structure. At lower CRs, PLLA/PLLA-g-IA/ATBC/SCG reached the highest values of the degree of crystallinity (X_c_). Thus, the study of crystallization kinetics showed that SCG significantly increased the crystallization rate in all studied materials. In terms of thermal stability, SCG reduced the initial temperature of degradation. However, the chemical reaction of the fatty acids contained in SCG with the ester groups of PLLA was partially prevented by grafting IA onto its backbone. It also had a positive effect on the formation of a supramolecular structure by increasing the X_c_. The same effect was achieved by the presence of an ATBC plasticizer. The combination of the abovementioned modifications resulted into highest X_c_ values while maintaining the desirable properties of the extruded films, such as high elongation at break. A positive effect of SCG on the overall ductility and the damping properties was also demonstrated by DMA analysis and an increase in the imaginary component of the dynamic modulus of elasticity.

## Figures and Tables

**Table 1:** Masterbatch compositions.

Samples	PLLA (g)	DCP (g)	IA (g)	ATBC (g)	SCG (g)
MB1	50.00	0.85	10.20	0	0
MB2	50.00	0.85	10.20	0	17.00
MB3	74.95	0	0	17.00	0
MB4	60.00	0	0	0	17.00

**Table 2:** Sample compositions.

Samples	PLLA (wt.%)	IA (wt.%)	ATBC (wt.%)	SCG (wt.%)
PLLA	100	0	0	0
PLLA/SCG	90	0	0	10
PLLA/PLLA-g-IA	94	6	0	0
PLLA/PLLA-g-IA/SCG	84	6	0	10
PLLA/ATBC	90	0	10	0
PLLA/ATBC/SCG	80	0	10	10
PLLA/PLLA-g-IA/ATBC	84	6	10	0
PLLA/PLLA-g-IA/ATBC/SCG	74	6	10	10

**Table 3:** The results from the second heating run of DSC analysis.

Samples	CR (°C/min)	T_g_ (°C)	T_m_ (°C)	T_pc_ (°C)	T_sc_ (°C)	ΔH_m_ (J/g)	ΔH_pc_ (J/g)	ΔH_sc_ (J/g)	X_c_ (%)
PLLA	5	60.9	175.5	160.9	113.0	40.2	1.4	25.9	12.2
	10	60.3	174.7	160.4	111.9	39.1	0.5	32.2	6.0
	20	60.9	175.5	160.9	113.0	38.2	0.3	33.3	4.4
	40	60.9	175.7	156.1	113.0	38.4	0.3	34.4	3.5
PLLA/SCG	5	-	173.9	-	-	43.4	0.0	0.0	45.5
	10	-	173.6	160.3	-	41.2	1.8	0.0	41.3
	20	59.0	172.5	155.3	94.1	46.5	6.6	15.8	25.4
	40	58.8	172.4	155.3	97.2	45.9	6.7	26.7	13.1
PLLA/PLLA-g-IA	5	59.6	172.4	156.1	105.1	44.6	4.5	8.6	31.7
	10	58.9	172.7	157.2	108.2	41.6	1.8	28.7	11.1
	20	59.4	173.1	158.6	109.2	41.2	1.0	33.7	6.4
	40	59.3	173.1	158.6	109.4	40.4	1.0	35.0	4.4
PLLA/PLLA-g-IA/SCG	5	-	172.6	159.6	-	39.7	1.3	0.0	43.1
	10	-	172.0	155.5	94.8	43.1	5.0	4.1	38.2
	20	59.1	171.7	154.9	99.2	43.7	5.7	24.9	14.6
	40	59.0	171.7	155.1	99.2	43.3	5.5	28.3	10.6
PLLA/ATBC	5	-	169.2	153.1	-	40.4	1.5	0.0	40.8
	10	-	169.1	147.2	86.6	42.5	5.2	10.2	28.4
	20	40.8	168.9	148.9	92.7	42.3	4.4	24.2	14.3
	40	40.1	168.9	149.3	93.0	42.2	4.1	27.5	11.2
PLLA/ATBC/SCG	5	-	169.8	-	-	38.5	0.0	0.0	45.4
	10	-	169.3	154.9	-	38.5	0.8	0.0	44.5
	20	-	169.0	145.7	77.1	42.0	5.8	5.6	36.1
	40	40.9	168.7	143.9	82.5	42.3	6.5	18.6	20.4
PLLA/PLLA-g-IA/ATBC	5	-	170.0	153.1	-	37.5	1.4	0.0	40.6
	10	-	169.1	146.7	90.1	40.5	4.9	12.0	26.5
	20	43.9	169.5	148.9	94.5	39.2	4.1	23.7	12.8
	40	43.6	169.4	149.1	94.4	39.1	3.7	26.1	11.0
PLLA/PLLA-g-IA/ATBC/SCG	5	-	171.5	-	-	37.7	0.0	0.0	48.0
	10	-	170.2	152.0	91.8	39.9	3.9	0.9	45.2
	20	49.1	170.2	149.3	91.3	42.5	5.9	19.2	22.2
	40	49.1	170.2	148.9	91.4	42.3	5.9	23.8	16.1

**Table 4:** The results from the cooling run of DSC analysis.

Samples	CR (°C/min)	T_c_ (°C)	ΔH_c_ (J/g)	ΔH_Tc_ (J/g)	X_T_ (%)
PLLA	5	101.7	5.43	2.40	44.2
	10	100.2	1.18	0.48	40.7
	20	-	-	-	-
	40	-	-	-	-
PLLA/SCG	5	108.2	35.45	20.13	56.8
	10	105.7	33.35	17.22	51.6
	20	96.2	12.20	5.97	48.9
	40	93.7	0.81	0.44	54.3
PLLA/PLLA-g-IA	5	100.9	21.94	9.60	43.8
	10	96.6	3.29	1.61	48.9
	20	-	-	-	-
	40	-	-	-	-
PLLA/PLLA-g-IA/SCG	5	105.9	33.37	17.87	53.6
	10	99.3	23.48	10.91	46.5
	20	94.5	2.67	1.46	54.7
	40	91.7	0.10	0.06	60.0
PLLA/ATBC	5	93.8	31.48	17.95	57.0
	10	86.6	16.97	8.22	48.4
	20	87.5	1.37	0.58	42.3
	40	-	-	-	-
PLLA/ATBC/SCG	5	101.6	33.17	22.33	67.3
	10	94.2	29.35	18.14	61.8
	20	86.9	17.34	8.21	47.3
	40	83.7	0.99	0.53	53.5
PLLA/PLLA-g-IA/ATBC	5	95.3	28.86	16.02	55.5
	10	88.6	11.92	5.55	46.6
	20	88.1	1.24	0.50	40.7
	40	-	-	-	-
PLLA/PLLA-g-IA/ATBC/SCG	5	101.9	31.84	18.27	57.4
	10	95.0	25.47	12.36	48.5
	20	90.9	4.21	1.91	45.4
	40	-	-	-	-

**Table 5:** The modified Avrami parameters for the non-isothermal crystallization of PLLA, PLLA/PLLA-g-IA, PLLA/ATBC, and PLLA/PLLA-g-IA/ATBC composites.

Samples	CR (°C/min)	t_1/2_ (min)	log Z_t_	Z_c_	n	r^2^
PLLA	5	3.87	−2.10	0.00159	3.25	0.99
	10	2.35	−1.70	0.00201	3.98	0.99
	20	-	-	-	-	-
	40	-	-	-	-	-
PLLA/SCG	5	3.23	−2.46	0.00069	4.58	0.98
	10	2.03	−1.57	0.00267	4.75	0.98
	20	1.25	−0.58	0.01315	3.86	0.99
	40	0.34	1.18	0.37839	2.71	0.98
PLLA/PLLA-g-IA	5	4.43	−2.72	0.00038	3.84	0.99
	10	2.50	−1.83	0.00148	3.71	0.97
	20	-	-	-	-	-
	40	-	-	-	-	-
PLLA/PLLA-g-IA/SCG	5	4.42	−3.07	0.00017	4.55	0.96
	10	2.97	−2.03	0.00094	3.81	0.96
	20	1.70	−1.08	0.00416	4.05	0.98
	40	0.35	1.19	0.38720	2.63	0.96
PLLA/ATBC	5	5.32	−2.92	0.00024	3.83	0.99
	10	3.79	−2.06	0.00088	3.50	0.99
	20	1.18	−0.54	0.01442	4.06	0.99
	40	-	-	-	-	-
PLLA/ATBC/SCG	5	4.65	−3.13	0.00015	4.56	0.95
	10	2.54	−1.64	0.00228	4.18	0.96
	20	1.75	−1.05	0.00446	3.22	0.95
	40	0.40	1.05	0.28050	2.96	0.99
PLLA/PLLA-g-IA/ATBC	5	4.54	−2.71	0.00039	3.93	0.99
	10	3.10	−1.86	0.00138	3.57	0.99
	20	1.11	-0.38	0.02084	3.64	0.99
	40	-	-	-	-	-
PLLA/PLLA-g-IA/ATBC/SCG	5	4.37	−2.88	0.00027	4.25	0.97
	10	3.18	−2.23	0.00059	4.01	0.96
	20	1.34	−0.67	0.01069	3.79	0.99
	40	-	-	-	-	-

**Table 6:** Thermodegradation temperatures.

Samples	T_d,5_ (°C)
PLLA	328
PLLA/SCG	310
PLLA/PLLA-g-IA	337
PLLA/PLLA-g-IA/SCG	316
PLLA/ATBC	272
PLLA/ATBC/SCG	272
PLLA/PLLA-g-IA/ATBC	272
PLLA/PLLA-g-IA/ATBC/SCG	279

**Table 7:** Tensile modulus, strength, and elongation at break of PLLA-based films.

Samples	E_t_ (MPa)			σ_m_ (MPa)			ε_tb_ (%)		
	$\bar{x}$	s	CI(95)	$\bar{x}$	s	CI(95)	$\bar{x}$	s	CI(95)
PLLA	3530	193	120	50	3	2	8	1	1
PLLA/SCG	3599	222	138	36	2	1	19	4	2
PLLA/PLLA-g-IA	3603	173	107	46	2	1	4	1	1
PLLA/PLLA-g-IA/SCG	3386	221	137	33	2	1	11	4	2
PLLA/ATBC	2834	300	186	39	2	1	7	3	2
PLLA/ATBC/SCG	2049	193	120	25	1	1	36	11	7
PLLA/PLLA-g-IA/ATBC	1475	135	84	25	2	1	127	88	55
PLLA/PLLA-g-IA/ATBC/SCG	1293	267	165	23	3	2	241	35	22

**Table 8:** Values of storage modulus, loss modulus, complex modulus, and loss factor.

Samples	T (°C)	E′ (MPa)	E″ (MPa)	tan δ (-)	E* (MPa)
PLLA	10	2276	37	0.016	2277
	23	2222	42	0.019	2223
	40	2140	51	0.024	2141
	50	2088	65	0.031	2090
PLLA/SCG	10	2632	43	0.017	2633
	23	2596	44	0.017	2597
	40	2465	56	0.023	2466
	50	2354	76	0.032	2356
PLLA/PLLA-g-IA	10	2449	35	0.015	2449
	23	2374	45	0.019	2375
	40	2260	58	0.026	2261
	50	2147	80	0.038	2150
PLLA/PLLA-g-IA/SCG	10	3074	45	0.015	3074
	23	3012	48	0.015	3013
	40	2833	69	0.024	2834
	50	2617	139	0.047	2623
PLLA/ATBC	10	2282	65	0.028	2284
	23	2251	63	0.028	2253
	40	2017	90	0.044	2021
	50	-	-	-	-
PLLA/ATBC/SCG	10	2009	185	0.093	2027
	23	1907	181	0.096	1925
	40	1412	238	0.169	1453
	50	-	-	-	-
PLLA/PLLA-g-IA/ATBC	10	2506	133	0.053	2513
	23	2361	144	0.061	2370
	40	1007	384	0.038	1131
	50	-	-	-	-
PLLA/PLLA-g-IA/ATBC/SCG	10	2733	122	0.045	2738
	23	2516	133	0.053	2523
	40	1131	376	0.033	1241
	50	-	-	-	-

## Data Availability

Not applicable.
